# Global collaboration between platform trials in surgery and anaesthesia

**DOI:** 10.1093/bjs/znae262

**Published:** 2024-12-20

**Authors:** James Glasbey, Steve A Webb, Trisha Peel, Thomas D Pinkney, Paul S Myles

**Affiliations:** Academic Department of Surgery, University of Birmingham, Birmingham, UK; Australian and New Zealand Intensive Care Research Centre, School of Public Health and Preventive Medicine, Monash University, Melbourne, Victoria, Australia; Director of Research, St John of God Healthcare, Melbourne, Victoria, Australia; Department of Infectious Diseases, Monash University, Melbourne, Victoria, Australia; Department of Infectious Diseases, Alfred Hospital, Melbourne, Victoria, Australia; Academic Department of Surgery, University of Birmingham, Birmingham, UK; Department of General Surgery, University Hospitals Birmingham NHS Foundation Trust, Birmingham, UK; Department of Anaesthesiology and Perioperative Medicine, Monash University, Melbourne, Victoria, Australia; Department of Anaesthesiology and Perioperative Medicine, Alfred Hospital, Melbourne, Victoria, Australia

**Keywords:** adaptive trials, anaesthesia, collaborative research, perioperative care, platform trials, surgery, surgical site infection

## Abstract

Large, randomized trials are the bedrock of evidence-based medicine, but the resources required to complete such trials greatly limit the number of important clinical questions that can be addressed within a reasonable period of time. Adaptive platform trials can identify effective, ineffective, or harmful treatments faster. These trials have been shown to deliver rapid evidence through the COVID-19 pandemic and are now being adopted across surgery and anaesthesia, with many opportunities for surgeons, anaesthetists, and other perioperative physicians to conduct and collaborate in platform trials.

*‘A platform trial is an engine room for multiple clinical trials in parallel and series. Trials are done faster and cheaper, with capacity for near-immediate translation into clinical practice’*

*Paul Myles 2022*

There are many potentially effective (or harmful) interventions in surgery and anaesthesia, but there are too few opportunities to evaluate all of these in large-scale clinical trials^1^. There is substantial between-patient variation in the interventions that lie within the spectrum of standard care. Insufficient evidence about effectiveness, comparative effectiveness, and cost-effectiveness facilitates variation and likely contributes to variation in patient outcome and health system productivity. Innovations in trial design offer solutions^2^.

The term ‘platform trial’ broadly describes a flexible, adaptive design that allows researchers to answer more than one primary research question and add new questions within the same master protocol^3–6^. Platform trials can evaluate multiple interventions both alone and in combination against one or more control groups, offering the opportunity to optimize treatment for a disease area, rather than evaluating a single health technology (*Fig. 1*). Any chosen control group is most often determined by contemporaneous randomization for the intervention(s) under study at that time, thus minimizing temporal trends. The design facilitates interim analyses, which allow interventions (or combinations) with overwhelming evidence of benefit (that is, a superiority trigger), low likelihood of benefit or with evidence of harm (that is, a futility trigger) to be stopped early. Thus, the master protocol outlines how domains or treatments within a domain under study, including future additional treatments, can leave or enter the platform during the lifetime of the trial. This increases overall efficiency and can be combined with methods by which more patients are assigned to interventions that are more likely to demonstrate benefit. New randomized comparisons (domains) can be added to a platform, and the standard of care within the control group can be actively updated as results emerge, thereby improving practice directly and contemporaneously via the research platform. Other adaptive elements that can be modified include eligibility criteria, required sample size for any comparison(s), randomization assignment ratio, and the addition of other promising treatments or treatment combinations^4,5^.

**Fig. 1:**
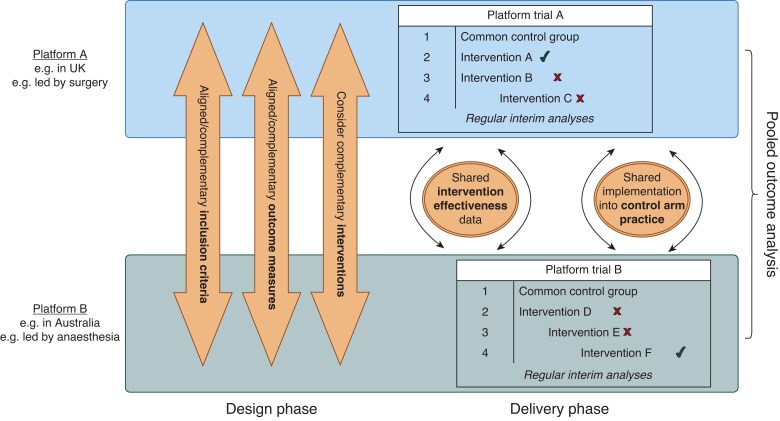
Conceptual overview of collaboration between platform trials A common control group may include the entire control cohort across the lifecycle of the trial (both ‘non-concurrent’ and ‘concurrent’ control groups) or be a subset that was enrolled and randomized in the same time interval as the particular treatment being evaluated (‘concurrent control group’). The tick indicates randomization to an intervention stopped after demonstrating superiority. The cross indicates randomization to an intervention stopped after demonstrating futility or harm.

The statistical design of platform trials can be informed using a frequentist approach^7,8^, as in the STAMPEDE trial in prostate cancer, and ROSSINI 2 trial in surgical wound healing^9^, or a Bayesian approach^10^, such as in the REMAP-CAP trial in community-acquired pneumonia^10^. Platform trials bring multiple levels of efficiency for patients, researchers, and policy makers^11,12^. They are administratively efficient in sharing a single master protocol, leadership structure, site initiation, and ethical or governance approval processes. They are cost-efficient in bringing economies of scale to central trial administration, follow-up procedures, data collection, and intervention supply and distribution. They are also statistically efficient by using information about differences in observed effects between combinations of interventions to gain additional information about the overall effect of a single intervention. Trials of bundled interventions can be used to test multiple interventions concurrently, but these cannot evaluate the effectiveness of individual components and risk misinforming clinicians^13^.

## Perioperative platform trials

Both surgery and anaesthesia research are ideal areas within which to apply platform designs, for several reasons. First, patients who undergo an operation are an easy group to define, identify, and screen. Second, they have a substantial risk of adverse events, many of which could be modifiable, and can usually be measured within days, weeks, or perhaps months after surgery^14^. This allows outcome data to be rapidly accrued for use in interim analyses with or without adaptive randomization. Third, innovation and practice variation are common. Efficient designs are required to rapidly test multiple interventions (which may also interact with each other) to support implementation of beneficial and de-implementation of harmful practices.

Surgery itself is a complex intervention. It is unlikely that a single ‘magic bullet’ exists to prevent any postoperative complication; the risk of adverse events is multifactorial and complex, and reflects individual patient characteristics, the biology of disease, operation, and the aggregate effect of decisions in relation to components of anaesthesia and surgery that occur before, during, and after the surgical procedure. One such example is surgical-site infection, which is typically measured up to 30 days after surgery^15–17^. Four platform trials have emerged in surgical-site infection prevention taking complementary surgical and anaesthetic perspectives from research groups in the UK (ROSSINI-2: NCT03838575, ROSSINI-Platform), a collaborating network of seven low- and middle-income countries (MARLIN: NCT06465901), and Australasia (PROMPT). This presents a unique opportunity for shared operational, methodological, and clinical learning, which have not previously been explored.

## Collaboration between platforms in wound infection prevention

Trials with the same primary outcome and with similar eligibility criteria are often considered to be ‘competing’^18^. However, considering platform trials as such would miss a critical opportunity to benefit both patients and research teams. During the 2024 Anaesthesia Platform Trials Symposium at the Monash Centre in Prato, Italy, a memorandum of understanding was agreed to collaborate between these trials over their duration. At a high level this involved considering them as decentralized, federated platforms^19^. This allows teams to develop, lead, and deliver their separately funded and powered platforms independently, but with agreement to harmonize key design considerations, including harmonization of entry criteria, interventions, and outcome measures, and introduce opportunities for shared learning and data pooling to more rapidly inform evidence-based perioperative practice.

The perioperative setting offers many other outcomes that could be a focus for platform trials, including postoperative delirium, respiratory, thromboembolic and cardiovascular complications, and unplanned hospital readmission.

Proposed areas for collaboration include:

- Patients: Ensuring similar, pragmatic eligibility criteria and definitions of descriptive data to allow direct comparisons and pooling of individual participant data.
- Interventions: Transparently reporting intervention selection criteria for evaluation. Sharing early results of interim and final analyses to inform the addition of complementary intervention arms (for example re-evaluation in a different patient group).
- Comparators: Harmonizing monitoring of ‘standard practice’ with pragmatic platform trials to increase contextual understanding of evaluation and inform discussions around heterogeneity of treatment effects observed.
- Outcomes: Sharing outcome definitions and processes for assessment to support seamless translation of knowledge and data pooling between platforms. Identify opportunities to evaluate important but rarer endpoints through data sharing.
- Monitoring and governance: Considering shared oversight committee members and sharing patient information materials and ethics documents for local adaptation.
- Methodology: Sharing novel methodological insights and addressing emerging design and analysis questions together to improve generalizability.
- Conduct: Combining and documenting practical tips and lessons learnt to inform the wider global trials community.
- Health and carbon economics: Harmonizing perspectives, costings, models, and life cycle assessments to inform within-trial cost and carbon equivalent (eCO_2_) effectiveness analyses, and share lessons around contingent outcomes based on these important secondary considerations.
- Funding: Exploring options for joint funding to expedite evaluation of promising emerging health technologies.

## Conclusions

Twenty years ago, surgery and anaesthesia were the specialty areas with the lowest rate of recruitment to clinical trials. This was reflected in Horton’s infamous *Lancet* editorial, which shined a light on surgical care as the problem child of evidence-based medicine, with practitioners that ‘lack the interpersonal attributes that are needed for multicentre collaborative research’^20^. Initiatives such as the Royal College of Surgeons (RCSEng) clinical trials initiative in the UK, the Australian and New Zealand College of Anaesthetists Clinical Trials Network (ANZCA CTN), and the NIHR Global Health Research Unit on Global Surgery (GSU) have fostered the resurgence of high-quality clinical trials for patient benefit in perioperative care^21^. This new agreement highlights surgery and anaesthesia together at the cutting edge of collaborative science. It represents a clear shift from the traditional consideration of ‘competing trials’ into a future vision for ‘collaborating trials’ that can optimize whole pathways of perioperative care as part of routine practice. Platform trials are highly efficient, with the potential to provide quicker answers to important clinical questions that lead to improved patient care. Researchers working on platform trials with the same primary outcome in other disease areas can consider these insights in informing future collaborations.
